# Standard-Setting of Multidisciplinary Objective Structured Practical Examination

**DOI:** 10.7759/cureus.25296

**Published:** 2022-05-24

**Authors:** Sherif M Zaki, Amira S Ismail

**Affiliations:** 1 Anatomy, Fakeeh College for Medical Sciences, Jeddah, SAU; 2 Medical Education, Suez Canal University, Ismailia, EGY

**Keywords:** standard setting, medical students, modified ebel's methods, multidisciplinary objective structured practical examination, musculoskeletal and integument systems module

## Abstract

Purpose

The present work applies the standard-setting in the multidisciplinary Objective Structured Practical Examination (OSPE) of the MusculoSkeletal and Integument systems (MSK) module using Modified Ebel’s method to differentiate the competent students from the non-competent ones.

Materials and methods

One hundred fifty-six students participated in the multidisciplinary OSPE. The MSK-OSPE consists of mid-module and final. According to the blueprint of the OSPE, the mid-module OSPE tested the knowledge and skills of the upper limb, and the final OSPE verified the knowledge and skills of the lower limb. Modified Ebel’s method was used to identify the Minimum Pass Level (MPL) in each station and the whole exam accordingly.

Results

Fifty-seven percent (57%) of the students passed both exams, while 25.6% did not pass the mid-module exam and 31.4% did not pass the final exam, 17.9% did not pass both exams and 25% did not pass one exam. The MPL for most of the stations in both exams using modified Ebel’s method of the standard-setting was more than 50% which is the conventional pass mark. However, the MPL for stations 4, and 6 in the mid-module exam (ulna and arteries of the upper limb) and stations 7, 9, and 14 (muscles of the lower limb, anatomy of ankle joint, physiology of nerve) was < 50%. While the total pass mark of the mid-module OSPE was 66% and the pass mark for the final OSPE was 60%

Conclusion

The minimal pass level (MPL) in mid module and final OSPE were 66% and 60% respectively which are more than the conventional cut off point (50%) that indicating that the standard-setting was effective in identifying poor performers who cannot be identified by the conventional method that led to enhance the quality of OSPE as an assessment tool. Moreover, students developed the skills to deal with standardized patients in clinical stations. However, some defects and areas of improvement were identified in some physiological and anatomical stations. The organizing committee recommended identifying the poor performers and conducting extra-tutorial sessions on the defective topics.

## Introduction

Most medical schools assess the practical parts through the standard spotter tests and objective structured practical examination (OSPE) [1]. Some schools use the oral (viva voce) examination [1]. OSPE is the widespread method of assessing practical basic science in the early phase of the medical curriculum [2]. OSPEs evaluate the theoretical, applied, and technical skills instantaneously [3,4]. OSPEs differ from standard spotter examinations in many aspects. The station in OSPE has one specific objective, tests a higher level of the knowledge domain, and is structured according to the exam's blueprint [5].

Two different disciplinary OSPEs are usually used to assess the students, the classical multi-disciplinary OSPE and mono-disciplinary OSPE. Most institutes use the mono-disciplinary OSPE where the examination runs in one discipline [6]. Fakeeh College for Medical Sciences is a newly developing private college that offers four undergraduate programs. One of them is the Bachelor of Medicine, Bachelor of Surgery program (MBBS). The MBBS program is a system-based, spiral-integrated program. The program is delivered as modules in the preclinical phase, each module has theoretical and practical examination. The program utilized the classic multi-disciplinary OSPE as this type of examination allows the integration between different basic science and clinical sciences.

The musculoskeletal and Integument system (MSK) module commenced in the academic year 2018-2019. The 2nd year medical undergraduates undertake this module. The module is four credit hours, delivered in five weeks and divided into two theoretical hours and two practical hours.

Based on the course specification of the module, the practical assessment of the MSK module is organized through two multidisciplinary OSPEs (mid-module and final). The interval period between the two examinations is three weeks. The current study aimed to apply the standard-setting using Modified Ebel’s methods to the multidisciplinary OSPE to ensure high-quality OSPE as an assessment tool differentiating between competent and non-competent students.

## Materials and methods

Design

The current study was a descriptive observational study.

Sampling

The sample was comprehensive, with 156 students (100 female, 56 male) in the 2nd year enrolled in the current study. All students completed the MSK course and participated in the multidisciplinary OSPE.

Description of multidisciplinary OSPE

Based on the course specification of the MSK module, the module has two multidisciplinary OSPEs (mid-module and final). Both OSPEs were conducted in May 2021. the examinations were designed in alignment with the intended learning outcomes of the module. The OSPE stations were assessed by a multidisciplinary faculty committee and an Examination Reliability analysis was conducted.

The organizing committee was formulated of thirteen members, two members from each discipline and one medical educationist. The disciplines represented in this committee are Anatomy, Physiology, Histology, Pathology, Radiology, and Orthopedic. All committee members are Ph.D. holders and highly qualified in their area of expertise with more than three years of teaching experience. In addition to that, all the committee members were familiar with the students and their performance. The questions were created following the blueprint [7]. The answer key was prepared before the examination.

**Figure 1:**
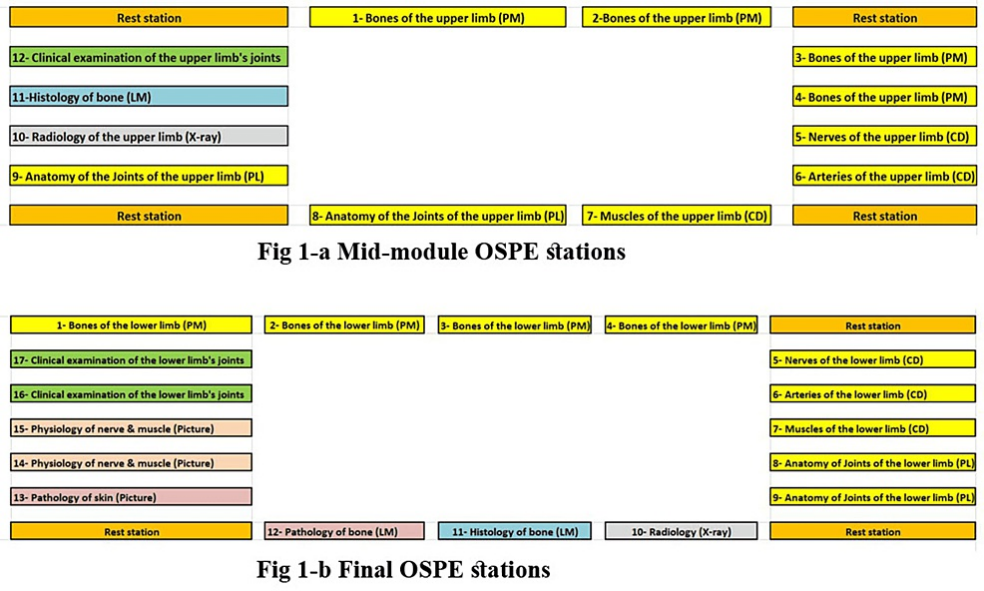
Mid-module and final OSPE stations

There were 12 stations in the mid-module OSPE and 17 stations in the final OSPE. The time allowed in each station was 3-5 minutes. Each station was marked out of 5. In alignment with the integrated nature of the MBBS program, the multi-disciplinary OSPE included nine anatomical, one histological, one radiological, and one clinical examination (Figure 1a).

**Figure 2:**
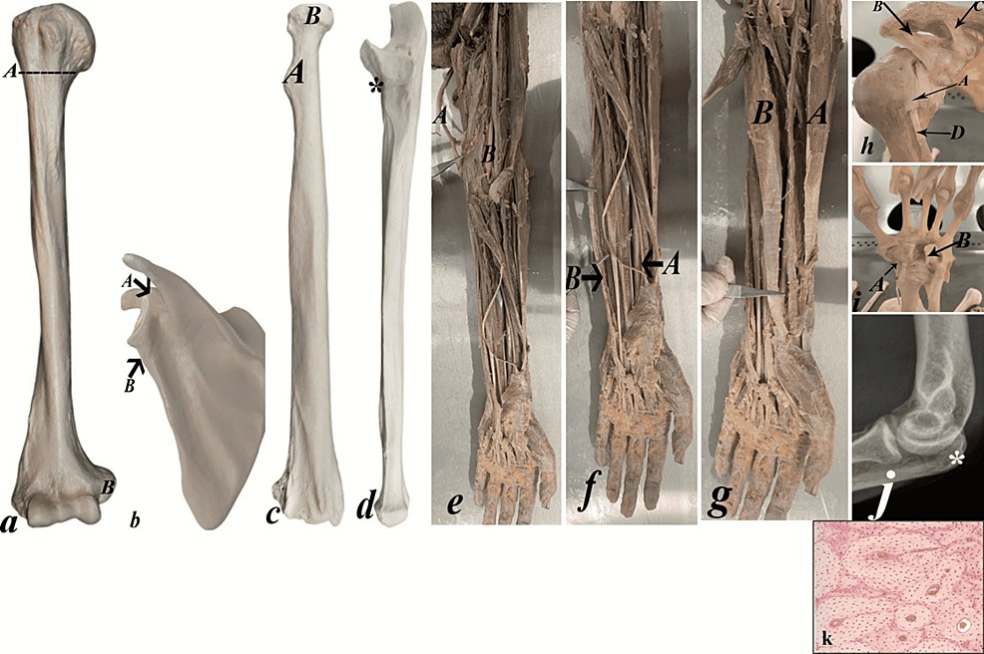
The detailed questions and figures of each station in mid-module and final OSPE

**Figure 3:**
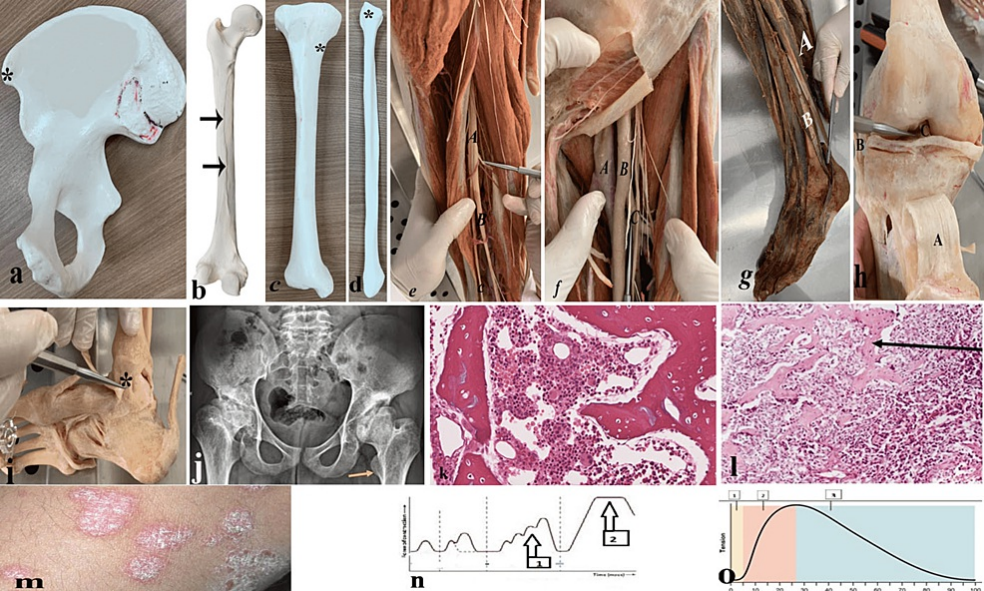
The detailed questions and figures of each station of mid-module and final OSPE

While, the final OSPE stations were nine anatomical, one histological, one radiological, two physiological, two pathological, and two clinical examinations (Figure 1b). The detailed questions and figures of each station were provided below (Figures 2, 3) and (Tables 1, 2).

**Table 1:** Mid-module stations

Station	Fig	Case scenario	Task
1	2-a	A 20-year-old woman was diagnosed with a fractured surgical neck of the humerus	1- Which one of the marked structures was affected (A or B)? --------- A. 2- Name the affected nerve------Axillary nerve.
2	2-b	The MRI examination of the shoulder of a 62-year-old female demonstrated erosion of the tendon of the long head of the biceps.	1- Which one of the marked structures gives attachment to the eroded tendon (A or B)? Mention its name----A, supraglenoid tubercle. 2- Name the nerve supply of the affected muscle—The musculocutaneous nerve.
3	2-c	X-ray examination of the elbow region of a 50 -year-old male displayed radial head dislocation.	1 - Which one of the marked structures was affected in this case (A or B)? --A 2- Name two joints shared by the displaced head? ------elbow, superior radioulnar joints.
4	2-d	The MRI examination of the elbow region of a 50 -year-old male displayed a tear of the tendon attached to the marked area (*).	1- Name the marked area. What is the affected muscle? -----Ulnar tuberosity, brachialis. 2- Name the action of the affected muscle-------Flexion of the elbow joint.
5	2-e	A 20-year-old woman presents in the outpatient clinic with a partial claw hand deformity.	1- Which one of the marked nerves was affected (A or B)? Mention its name ----------- A, ulnar nerve. 2- Name two muscles supplied by the affected nerve ---3^rd^ & 4^th^ lumbricals.
6	2-f	A 16 -year-old student presented in the ER with a cut wrist. The ulnar artery was injured.	1- Which one of the marked arteries was affected in this case (A or B)? B 2- Name two branches given by the affected artery-- Anterior & posterior ulnar recurrent.
7	2-g	A 20-year-old woman presents with ape hand deformity two months after an accident in the elbow region.	1- Which one of the marked muscles is paralyzed (A or B)? Mention its name---------B, Flexor carpi radialis. 2- Name actions of the marked muscle----Flexion, abduction of the hand.
8	2-h	A 23-year-old male presents to the ER with a shoulder injury. Examinations revealed total separation of the shoulder with the affection of the marked ligament.	1- Name the marked ligament----------Transverse humeral ligament 2- Name the attachment of the affected ligament--------It extends between the greater and lesser tuberosities.
9	2-i	A 34-year-old female complains of tingling of the palmar aspect of her right thumb, index, and middle fingers. She is diagnosed with carpal tunnel syndrome.	1- The surgeon must split one of the following marked structures (A or B) to relieve the presenting symptoms? Name its name-----B, Flexor retinaculum. 2- Explain the sensory complaint------compression of the median nerve.
10	2-j	A 25-year-old male presents to the ER after he slipped. The ER physician requested imaging for a suspected bony fracture.	1- Identify the radiological study------Plain X-ray. 2- What is the marked suspected bony fracture (*)? Olecranon process
11	2-k	A 45-year-old male presents with a lamb in his lower limb. A frozen section is taken from the mass to exclude malignancy.	1- Identify the token tissue sample---- A section of compact ground bone. 2- What is the system seen under the light microscope? Haversian system.
12		A 44-year-old male presents to the orthopedic clinic with a 7- week history of right elbow pain radiating into the forearm. His symptoms started gradually and became worse.	You are requested to examine the patient's elbow and summarize the findings to him.

**Table 2:** OSPE final stations

Station	Fig	Case scenario	Task
1	3-a	MRI of a 62-year-old female confirmed erosion of the muscle attached to the marked area (*).	1- Name the marked area. What is the affected muscle? -----Anterior superior iliac spine, sartorius muscle. 2- Name the nerve supply of the affected muscle-------Femoral nerve.
2	3-b	X-ray examination of the thigh of a 20 -year-old male exhibited an open femoral shaft fracture. The MRI displayed injuries of the adductor muscles that are attached to the marked area.	1- Name the marked area-----------Linea Aspera. 2- Name the injured muscles-----Adductor longus, adductor brevis & pubic part of Adductor Magnus.
3	3-c	MRI examination of a 29-year-old athlete male revealed avulsion of the muscles attached to the marked bony area (*).	1- Name of the marked area---Upper part of the medial surface of the shaft of the tibia. 2- Name the avulsed muscles---- Sartorius, gracilis and semitendinosus.
4	3-d	MRI examination of the ankle region of a 50 -year- old male displayed a tear of the muscle & ligament attached to the marked area.	1- Name the marked area----- Head of the fibula. 2- Name the torn structures----- Biceps femoris & fibular collateral ligament.
5	3-e	A 20-year-old woman presents with drop foot deformity after a posterior dislocation of the hip joint.	1- Which one of the marked nerves is affected (A or B or C)? Mention its name ----------- A, the sciatic nerve. 2- Name two muscles directly supplied by the affected nerve----- Biceps femoris, semitendinosus, and semimembranosus.
6	3-f	A 16 -year-old student was seen in the ER with a stab wound injury to the inguinal region. The femoral artery was injured.	1- Which one of the marked vessels was affected in this case (A or B or C)? A 2- Name two branches given by the affected artery---Superficial epigastric, superficial external pudendal.
7	3-g	A 20-year-old woman was presenting with Talipes equino varus deformity after an accident in the knee region.	1- Which one of the marked muscles is paralyzed (A or B)? Mention its name -------- B, peroneus brevis. 2- Name actions of the marked muscle---Eversion, planter flexion of the foot.
8	3-h	A 20-year-old woman was diagnosed with a tear of the anterior cruciate ligament.	1- Which of the marked structures was affected in this case (A or B or C)? C 2- Name the function of the torn ligament---- It prevents hyperextension of the knee & prevents anterior dislocation of the tibia.
9	3-i	A 23-year-old male presents to the hospital after injuring his ankle joint. Physical and radiographic examinations revealed a tear of the marked ligament.	1- Name the torn ligament--------Deltoid ligament. 2- Name the proximal attachment of the affected ligament--- tip of the medial malleolus.
10	3-j	A 20-year-old male with known sickle cell anemia presents to the ER with decreased range of motion and painful movement. The following is the patient’s imaging.	1- What is your most likely diagnosis? ----- Avascular necrosis of the bilateral hip. 2- What is the structure pointed by the yellow arrow? --- Lesser trochanter.
11	3-k	A 45-year-old male presents with a lamb in the spine. A biopsy was taken to exclude malignancy.	Identify the token tissue sample---- Bone marrow
12	3-l	A 27-year-old patient is complaining of a rapidly growing mass in his right thigh. It was resected from the metaphysis of the femur. A biopsy for histopathological assessment was undertaken.	1- What is your diagnosis? ---------- Osteosarcoma. 2- Where the arrow is pointing? ---------------Neoplastic osteoid.
13	3-m	A 23-year-old man suddenly develops a gradually appearing rash over the trunk and extremities.	1- Describe the lesion----Erythematous papules, plaques and macules covered by white silvery scales. 2- What is your provisional diagnosis? ---Psoriasis.
14	3-n	A medical student used frog gastrocnemius –sciatic nerve preparation to obtain simple muscle twitch. He repeated stimulation of the nerve and he got the following curve.	1- Arrow 1 record is called------------ Treppe or staircase effect muscle. 2- Arrow 2 record is called-------------- wave stimulation and incomplete tetanus.
15	3-o	A group of medical students is doing lab experiments to demonstrate skeletal muscle contraction. Their instructor showed them the frog preparation that will be used to obtain a curve of simple muscle twitch.	Name the phases of simple muscle twitch shown (1,2,3). 1- Latent phase 2- Contraction period 3- Relaxation period
16		A 72-year-old female complains of pain in the right hip radiating to her knee. The pain is relieved by rest. She had a history of a slip and fall six years ago.	You are requested to examine the patient's hip and summarize the findings to the patient.
17		A 35- year- old male was playing football. He held his right foot steady on the floor and twisted his knee to push the football away.	You are requested to examine the patient's knee and summarize the findings to the patient.

The clinical examination stations tested the psychomotor skills in OSPE. These observed stations with standardized patients are very helpful to test skills of joint examination. The other stations included cadaveric specimens, plastic models, plastinated models, light microscopy, or images [5]. A clinical scenario was given at each station, and students were asked integrated questions. The questions were in two parts, the first part was the identification of the labeled structure, and the second part was relating that structure to its function [5]. Such a way of design tests the basic science competency [8].

Description of the standard-setting method (modified Ebel's method)

Modified Ebel’s method was used to identify the minimum pass level (MPL) of the OSPEs [9]. In this method, the subject matter expert of each discipline categorizes each station based on the difficulty level and relevance. An expected score at each station was calculated by multiplying the raw score for relevance and difficulty with the proportion of minimally competent students (Table 3).

**Table 3:** Calculation of the OSPE station's minimum pass level (MPL) with Modified Ebel's method.

Relevancy/Difficulty	Raw Score (A)	Proportion of borderline students who can answer correctly (B)	Expected Score= A x B x 100
Essential easy	1.0a	100	
Essential medium	0.9	90	
Essential difficult	0.8	80	
Important easy	0.7	70a	
Important medium	0.6	60	
Important difficult	0.5	50	
Marginal easy	0.4	40	
Marginal medium	0.3	30	
Marginal difficult	0.2	20	
		10	
Expected Minimum Passing Score (%)	(A x B) = MPL		

To set MPL for the whole mid-module and final OSPE, the average score of all stations was calculated by the organizing committee. The chi-square test was used to compare the number of students who scored less than pass marks in conventional methods and modified Ebel’s method.

Ethical considerations

The Institutional Review Board at Fakeeh College for Medical Sciences (FCMS) approved the study. The approval number is 43/IRB/2018. Permissions were taken from the undergraduate curriculum and examination committee.

## Results

The reliability analysis of mid- module and final multidisciplinary OSPE was performed, and the results were 0.834 in mid module and 0.880 in final OSPE which reflected very good reliability as shown in Table 4. In addition to that, all stations contributed to overall reliability in mid-module and final OSPE.

**Table 4:** Reliability analysis of mid-module and final OSPE

Examination	Cronbach's Alpha	No. of items
Mid-module OSPE	0.834	12
Final OSPE	0.880	17

The mid-module OSPE tested the knowledge, skills, and attitudes learning outcomes of the upper limb, and the final OSPE verified the learning outcomes of the lower limb. The MPL for the mid-module stations was as the following: stations 1-4 (anatomy of the bones) 93%,70%, 74%,41%; stations 5-7 (anatomy of the nerves, arteries, muscles) 64%, 34%, 59%; stations 8-9 (anatomy of the joints) 68 %, 58%; station 10 (X-ray station) 71%; station 11 (histological of the bone) 83%; and station 12 (clinical examination station) 82%. Accordingly, the pass mark of the mid-module exam was calculated to be 66%.

The MPL for the final exam stations was as the following: stations 1-4 (anatomy of the bones) 64%, 69%,52%, 55%; stations 5-7 (anatomy of the nerves, arteries, muscles of the lower limb) 55%, 63%, 39%; stations 8-9 (anatomy of the joints of the lower limb) 56 %, 48%; station 10 (X-ray station) 72%; station 11 (histological of the bone) 75%; stations 12-13 (pathology of bone, muscle and skin) 65 %, 53%; stations 14-15 (physiology of nerve and muscle) 31 %, 50%; and station 16-17 (clinical examination stations) 88% for both. Accordingly, the MPL for the final multidisciplinary OSPE was 60% (Table 5).

**Table 5:** MPL for the mid-module and final OSPE stations

OSPE	Station	MPL score station
Mid-module OSPE	Station 1 (Bones of the upper limb)	93
	Station 2 (Bones of the upper limb)	70
	Station 3 (Bones of the upper limb)	74
	Station 4 (Bones of the upper limb)	41
	Station 5 (Nerves of the upper limb)	64
	Station 6 (Arteries of the upper limb)	34
	Station 7 (Muscles of the upper limb)	59
	Station 8 (Joints of the upper limb)	68
	Station 9 (Joints of the upper limb)	58
	Station 10 (X-ray of the upper limb)	71
	Station 11 (Clinical examination of the upper limb’s Joints)	83
	Station 12 (Clinical examination of the upper limb’s Joints)	82
Final OSPE	Station 1 (Bones of the lower limb)	64
	Station 2 (Bones of the lower limb)	69
	Station 3 (Bones of the lower limb)	52
	Station 4 (Bones of the lower limb)	55
	Station 5 (Nerves of the lower limb)	55
	Station 6 (Arteries of the lower limb)	63
	Station 7 (Muscles of the lower limb)	39
	Station 8 (Joints of the lower limb)	56
	Station 9 (Joints of the lower limb)	48
	Station 10 (X-ray of the lower limb)	72
	Station 11 (Histology of the bone)	75
	Station 12 (Pathology of bone)	65
	Station 13 (pathology of skin)	53
	Station 14 (Physiology of nerves and muscles)	31
	Station 15 (Physiology of nerves and muscles)	50
	Station 16 (Clinical examination of the lower limb’s joints)	88
	Station 17 (Clinical examination of the lower limb’s joints)	88

The MPL for stations 4 and 6 in the mid-module exam (ulna and arteries of the upper limb) and stations 7, 9, and 14 (muscles of the lower limb, anatomy of ankle joint, physiology of nerve) was < 50%. No ambiguity was noted on the questions of these stations. So, these two stations were not dropped.

One hundred and fifty-six students (100 female, 56 male) were participating in the MSK module. All students completed the course. According to the conventional pass score, 25.6% (18.6% female,7.1% male) did not pass the mid-module exam. While 30.1% (21.8% female, 8.3% male) didn’t pass it using Modified Ebel’s methods. On the other hand, 25.5% (14.7% female, 10.8% male) did not pass the final exam. However, 31.4% (17.3%female, 14.1% male) failed the final by Modified Ebel’s method (Tables 6, 7)

**Table 6:** Mid-module and final OSPE results using conventional pass score

			Gender		Total
			Female	Male	
Mid-module OSPE	Not passing	Count	29	11	40
		% of Total	18.6%	7.1%	25.6%
	Passing	Count	71	45	116
		% of Total	45.5%	28.8%	74.4%
(Final OSPE	Not passing	Count	23	17	40
		% of Total	14.7%	10.8%	25.5%
	Passing	Count	77	39	116
		% of Total	49.5%	25%	74.5%
Both exams passing	Not passing both exams	Count	19	9	28
		% of Total	12.2%	5.8%	17.9%
	Not passing one exam	Count	22	17	39
		% of Total	14.1%	10.9%	25.0%
	Passing both exams	Count	59	30	89
		% of Total	37.8%	19.2%	57.1%
Total		Count	100	56	156
		% of Total	64.1%	35.9%	100.0%

**Table 7:** Mid-module and final OSPE results using Modified Ebel's method *pass mark 66%; **Pass mark 60%; ***p-value is insignificant

			Gender		Total	Chi-square test
			Female	Male		
Mid-module OSPE*	Not passing	Count	34	13	47	0.15***
		% of Total	21.8%	8.3%	30.1%	
	Passing	Count	66	43	109	
		% of Total	42.3%	27.6%	69.9%	
Final OSPE**	Not passing	Count	27	22	49	0.21***
		% of Total	17.3%	14.1%	31.4%	
	Passing	Count	73	34	107	
		% of Total	46.8%	21.8%	68.6%	
Both exams passing	Not passing both exams	Count	30	13	43	0.17***
		% of Total	19.2%	8.3%	27.6%	
	Not passing one exam	Count	19	18	37	
		% of Total	12.2%	11.5%	23.7%	
	Passing both exams	Count	51	25	76	
		% of Total	32.7%	16.0%	48.7%	
Total		Count	100	56	156	
		% of Total	64.1%	35.9%	100.0%	

The average pass scores in mid-module and final multidisciplinary OSPE using Modified Ebel's method were 66% and 60% accordingly. The OSPE organizing committee believed this achieved performance to be satisfactory. In addition to that, using the bell curve, the distribution of both OPSEs scores through applying modified Ebel’s method seems to take the normal distribution.

The percentage of students who scored >50% and >66% in mid-module OSPE were (74.4%) and (69.9%) respectively. This difference was significant (p < 0.001). In addition to that, The chi-square test showed that there is a significant difference between the percentage of passed students using the conventional pass mark (74.5%) and passed students percentage using Modified Ebel’s Method (68.6%) in the final OSPE (Table 7 above).

## Discussion

Traditional practical examination (TPE) was the basis of assessment of practical skills and knowledge for many years, and much was adjusted (MCQ, spot) to overcome the TPE flaws [10]. Recently, many authors favored the replacement of TPE with OSPE as OSPE has no inadequacies of the TPE, and deals with the shifting scenario of medical education, which entails problem-based learning, early clinical exposure, and inter-professional collaboration and education [6,11].

MBBS program at FCMS is a system-based, spirally integrated curriculum with an early clinical experience component, thereafter multidisciplinary OSPE was the aligned method of assessment utilized in such type of curriculum.

The musculoskeletal module is a multidisciplinary module where the teaching and learning process is delivered in an integrated manner. Thereafter the multidisciplinary OSPE is applied in this module. The main advantage of applying the multidisciplinary OSPE is that medical students should have a holistic picture of the disease starting from the anatomy, physiology, and pathology of the affected organ to the clinical aspect [8].

The mid-module OSPE assessed the learning outcomes of the upper limb, and the final OSPE verified the learning outcomes of the lower limb. The first nine stations assessed the anatomical knowledge and station 10 checked the applied radiological knowledge. We also analyzed pathological and physiological information in the final exam (stations 12-16) and the histology of bone and muscle in both exams. 

The mid module and final established an examination of joints (station 12 in the mid-module exam and stations 16 and 17 in the final exam) to assess psychomotor skills and verify the impact of anatomical knowledge on clinical skills. Schoeman and Chandratilake showed a weak relationship between anatomy competence and clinical skills among junior medical students [12]. However, in the current study, there was a good relationship between all the stations as reflected in the two OSPES’ reliabilities.

The reliability of mid-module and final OSPE were 0.83 and 0.88 respectively that reflecting very good internal consistency of the examination and this result comes in accords with a study conducted at Manipal University to assess the validity of OSPE in the pharmacology department [13].

Modified Ebel’s method was applied to the multidisciplinary OSPE and MPL is calculated for each station by subject matter experts, the MPL in mid-module and final ranged between 34 and 93. This result is similar to a study conducted at Al-Faisal University, Saudi Arabia [14]. This indicates that the multi-disciplinary OSPE stations have various difficulties and relevance.

There was statistical significance between the percentage of passed students using the conventional method and Modified Ebel’s method in mid-module and final OSPE. This result is consistent with the result of a study conducted in Melaka Manipal Medical College, India, aiming to set standards for OSPE [15]. This reflects that standard-setting methods help to differentiate between competent and incompetent students and this enhances the quality of OSPE as an assessment tool.

Feedback was sent to all participating departments to take all necessary actions for next year. The staff members in various departments advised more practical sessions for shutting any pertinent flaws.

Limitation of the study

The method was applied to the musculoskeletal module only. In addition to that, more than two experts are needed in each discipline.

## Conclusions

Teaching is an energetic process. We need constant assessment of our evaluation tools. The practical assessment of the MSK module is structured in two OSPEs (mid-module and final). The MPLs for mid-module and final multidisciplinary OSPE were 66% and 60% accordingly. The OSPE organizing committee believed this achieved performance to be satisfactory. The students developed the skills to deal with standardized patients through the clinical examination stations. Defects in some physiological and anatomical stations were observed. The organizing committee recommended identifying the poor performers and conducting extra-tutorial sessions on the defective topics.
